# Performance Evaluation of Mobile Liquid Cooled Thermoelectric Refrigeration System for Storage-Cum-Transportation of Fruits and Vegetables

**DOI:** 10.3390/foods11131896

**Published:** 2022-06-26

**Authors:** Prasad Chavan, Gagandeep Kaur Sidhu, Amit K. Jaiswal

**Affiliations:** 1 Department of Food Technology and Nutrition, Lovely Professional University, Phagwara 144402, India; erprasad.chavan@gmail.com; 2 Department of Processing & Food Engineering, Punjab Agricultural University, Ludhiana 141004, India; gagandeep@pau.edu; 3 School of Food Science and Environmental Health, Faculty of Science, Technological University Dublin-City Campus, Central Quad, Grangegorman, D07 ADY7 Dublin, Ireland; 4 Environmental Sustainability and Health Institute (ESHI), Technological University Dublin-City Campus, Grangegorman, D07 H6K8 Dublin, Ireland

**Keywords:** cold chain management, eco-friendly refrigeration, quality attributes, thermoelectric refrigeration, fruits and vegetables

## Abstract

The performance of a liquid-cooled thermoelectric refrigeration (LCTR) system for the storage of summer fruits and vegetables, viz., bitter gourd, okra, mango, and papaya, indicated notable results for physiological loss in weight, firmness, and colour values and overall acceptability of the crop. The LCTR system significantly reduced (*p* < 0.0001) the physiological loss in weight (PLW) of bitter gourd, okra, mango, and papaya to 11.51%, 10.99%, 12.29%, and 19.17%, respectively, compared to conventional ambient storage of the crop. A lesser change in colour was observed for the crop subjected to LCTR, recording 14.04, 11.46, 16.41, and 23.68 for bitter gourd, okra, mango, and papaya, respectively. All the crops witnessed no significant effect (*p* < 0.0001) on the quality attributes of the crop stored in LCTR and a vapour compression refrigeration system. LCTR enabled a pronounced increment in the shelf life of bitter gourd, okra, mango, and papaya by 7, 8, 10, and 13 days, respectively, compared to storage at ambient conditions. The invention provides a mobile thermoelectric refrigeration system useful for marketing fruits and vegetables efficiently. The system is economical, has a higher coefficient of performance (0.85) compared to the coefficient of performance (COP) of the existing thermoelectric refrigeration system, and maintains the freshness and quality of perishable agricultural produce during marketing and transportation.

## 1. Introduction

The global food production of the last 50 years has witnessed a significant increment of almost threefold in the face of a twofold increase in the global population. Despite this progress, one in eight people around the globe remain chronically undernourished [1]. In the Indian context, the per capita availability of fruits is limited to 107 g/day against the recommended level of 120 g/day [2]. Despite an impressive increase in production, post-harvest losses at various stages of supply chain management, which amount to 20–60% of the food produced across the world, have led to the uneven distribution and shortage of the qualitative food supply [2,3,4]. One of the major reasons for this food waste and food loss is the lack of an efficient cold chain infrastructure from the farm to the fork, which includes refrigerated transport, pack houses, collection centres, and cold storage. The major agricultural post-harvest losses accounted for in India were of the order of INR 9265.1 million [5], of which 4.58–15.88% was contributed by fruits and vegetables. Moreover, the physical loss of food has multiple consequences in association with the wastage of inputs such as water, fertilizer, electricity, human efforts, and precious time. To fulfil the global food demand, along with increasing food production, efficient supply chain management has to be adopted and the cold store preservation of fruits and vegetables from immediately after harvest to the end consumer has to be encouraged [6]. Cold chain management is the crucial operation that takes out heat energy from the food, and a lower temperature is maintained throughout the storage life of the food.

The basic principle of refrigeration preservation is to reduce the temperature of the food and maintain that temperature to a level such that the detrimental changes, such as the growth of microorganisms, ripening, browning reaction, pigment degradation, and moisture loss, are stopped or considerably reduced [7]. Cold preservation slows down not only the biological and chemical changes but also the physical and microbiological changes that cause spoilage of the food [8]. The modified atmosphere along with low-temperature storage controls the natural process of metabolism of the fruits and vegetables in conjunction with the reduced gas exchange through a barrier resulting in satisfactory shelf-life extension and the maintenance of the physicochemical constituents of produce [9,10,11]. The refrigeration system provides a very effective and efficient cold chain infrastructure to the freshly harvested commodities for maintaining freshness with an extended shelf life. As a result, it extends the storage life of food up to several weeks or a month without deteriorating the quality of fresh produce until it reaches the end consumer [12]. By way of maintaining the physicochemical and nutritional quality of food for a longer period, the cold preservation technique not only reduces the food losses in physical form but also lessens the economic losses to the manufacturer, as well as avoids the foodborne illness instigated by the multiplication of bacteria and microorganisms [13,14]. Thereby, the cold prevention technique maintains the availability of better-quality food produced in a distant market at a reasonable price while sustaining the circular food economy.

The chlorofluorocarbons (CFCs) and hydrochlorofluorocarbons (HCFCs) used as a refrigerant in the vapour compression refrigeration system contain chlorine, which causes ozone layer depletion; whereas, although hydrofluorocarbons (HFCs) are chlorine-free, these are green-house gases that cause the global temperature to rise. It has been reported that these gases have a global warming potential of a thousand times that of carbon dioxide [15]. According to United Nations Environment Program, refrigeration, air conditioning, and heat pumps annually contribute 40% of the CO_2_ equivalent emissions. It was estimated that almost 20% of the global warming impact was attributed due to the leakage of refrigerants into the atmosphere. An expert summit in 2009 at the Food and Agriculture Organization headquarters in Rome concluded that the food demand of the rising population can only be met when investment in scientific research and development will be followed along with the best environmental services to save the products from pests and preserve the perishable produce until it reaches the consumers’ table [16].

The thermoelectric refrigeration technique is a promising alternative because of its compactness, its silent working (as it does not involve a compressor), and because it has a wide range of temperatures [17,18,19]. The thermoelectric devices convert electricity into heat and vice-versa, with rigid structure, good stability, high reliability, and no evolution of greenhouse gas. The thermoelectric devices can be used for the development of thermoelectric refrigeration systems, which are based on the Peltier effect for removing heat by application of direct current (DC) across two dissimilar materials joined together, generating a temperature difference [20]. The thermoelectric refrigerator mainly consists of P and N-type semiconductors connected together, and when DC is applied across them, one junction becomes heated, and the other is cooled. Evidence for the applicability of the thermoelectric cooling principle is available from the cooling of sensors in aerospace, portable picnic coolers, automobiles, aircraft drinking water coolers, etc. Akinyemi and Simolowo [21] designed a small-scale mobile thermoelectric refrigeration system for the storage of beverages. Biswas and Kandasamy [22] developed a portable solar-powered thermoelectric cooler for the preservation of perishable foods, which can attain cold side temperatures up to 5 °C in 120 and 180 min without load and with loading (0.5 kg fish fillets), respectively. Rahman et al. [23] attempted to maximize the thermoelectric effect’s potential by harvesting heat rejected at the module’s hot side for low-temperature heating applications. The developed system could generate a cold temperature of 10 °C inside the cooling chamber, while maintaining a hot temperature of 40 °C. For both cooling and heating effects, experimental results showed a greater coefficient of performance (COP) of approximately 0.61. Efforts were also made by Ibikunle et al. [24] for the development of a 30 W single-stage thermoelectric cooler of 4-litre capacity that cools the vegetables from 27 to 5 °C in 3 h. However, no evidence is available for the commercial use of thermoelectric refrigeration for the preservation of horticultural crops.

Thus, in order to tackle the growing concern of food wastage due to the lack of scientific cold chain infrastructure for rural fruits and vegetable vendors and the simultaneous increasing demand for fresh fruits and vegetables, a thermoelectric refrigeration system was designed and developed [25]. The developed system has a larger storage capacity and a coefficient of performance (COP) of 0.85, which is higher as compared to other available systems. A study was undertaken to evaluate the performance of a developed thermoelectric refrigeration system. The performance was evaluated based on changes in the physical and mechanical properties and the shelf-life extension of fruits and vegetables stored in the system, and the results were compared with the vapour compression refrigeration system and ambient storage conditions.

## 2. Materials and Methods

### 2.1. Design of Thermoelectric Refrigeration System

**Figure 1:**
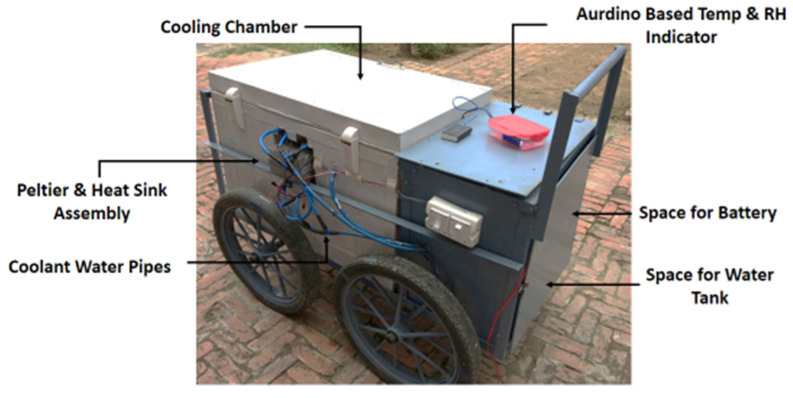
A complete view of the developed thermoelectric refrigeration system.

A battery-operated thermoelectric refrigeration system of 0.3 KW capacity was developed in the workshop of Punjab Agricultural University, Ludhiana. The cooling chamber of 100-L storage capacity (80 × 50 × 50 cm) was designed with a 6-cm thickness and the inside volume of the chamber was reduced to 100 L by partitioning. The main components of the developed thermoelectric refrigeration system include the cooling chamber, Peltier and heat sink assembly, coolant water pipes, coolant water storage tank, 12 V DC fan, fogger, temperature, and relative humidity indicator, as shown in Figure 1. Since the Peltier module required a DC, just as other electric appliances were operated on a DC, a 12 V electric battery was used in the system, and since the refrigerator was operated continuously, the battery was connected to the inverter for continuously recharging.

**Figure 2:**
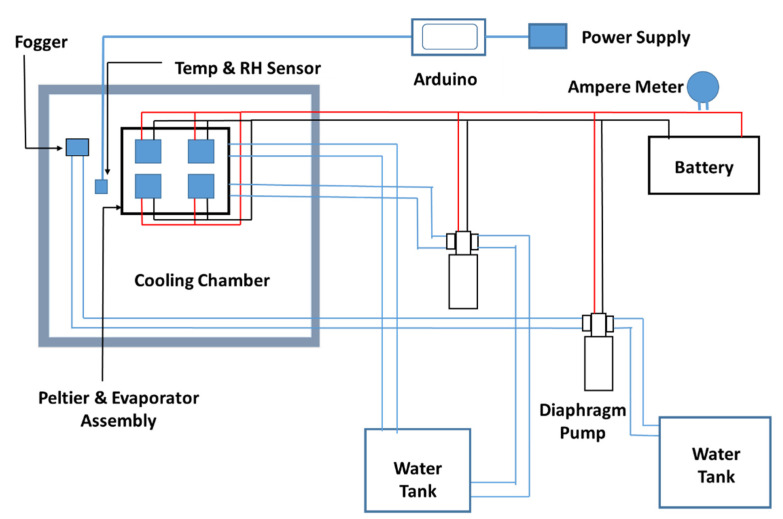
Schematic diagram of the component’s assembly of the thermoelectric refrigerator.

The assembly of the liquid-cooled thermoelectric refrigeration system consisted of four thermoelectric modules (TEC 12706 A), four aluminum liquid cooling heat sink blocks, and an elastomeric nitrile rubber sheet for insulation and two axial fans (12 V DC, 0.23 A). Two fin-type air-cooled heat sinks were assembled together facing side to side by riveting to make one heat sink of 20 × 15 × 8 cm. The cold sides of all (four) Peltier modules were pasted onto the cold side heat sink using thermal paste. Thermal paste is made of a polymerized liquid matrix of silicone of high thermal conductivity, which maintains a positive heat sink seal, facilitating efficient heat transfer from the electronic components to the heat sink. The hot side of each Peltier module was attached with a liquid-cooled sink. The clearance between the cold side and the hot side heat sinks was sealed using a 3-mm-thick elastomeric nitrile rubber insulation sheet, which presents the migration of heat from the hot side heat sink to the cold side heat sink. The inlet and outlet of all heat sinks were connected in parallel to the water tank through a plastic pipe of 1-cm diameter. Normal water was used as a coolant and circulated through evaporators using a diaphragm pump (12 V DC) powered by an electric battery. One fin-type air-cooled heat sink was attached over the liquid-cooled heat sink blocks, which supports the blocks as well as acts as an additional heat dissipation medium. The geometry of the liquid-cooled heat sink, connecting pipes, diaphragm pump, water tank, and the battery is shown in Figure 2.

**Figure 3:**
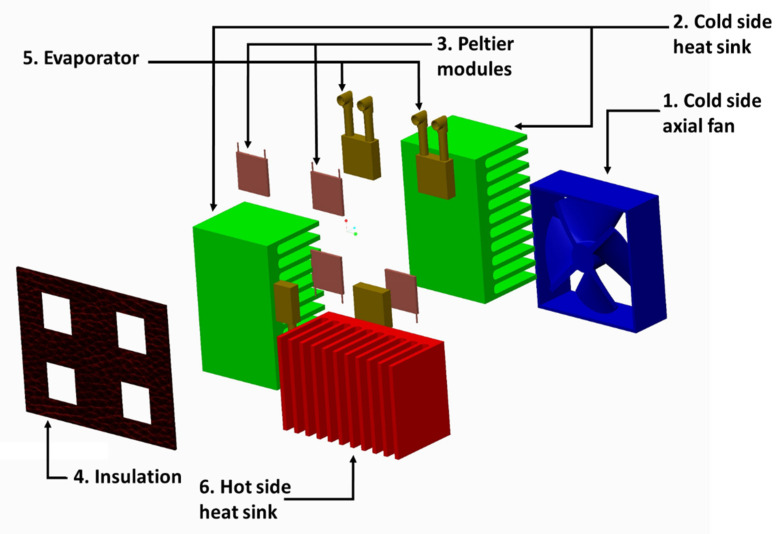
Expanded view of the liquid cooled heat sink assembly.

The computer-aided exploded view of the assembly of the heat sink, Peltier modules, and fan is shown in Figure 2. As thermoelectric modules were powered by a 12 V DC electric current, the heat sink attached to the cold side of the module becomes cooled and transfers heat to the hot side of the module. The axial fan attached to the cold side heat sink circulates cold air inside the cooling chamber, as presented in Figure 3. Water from the container is circulated through the water jackets attached to the hot side of the Peltier module. Water absorbs heat from the hot side and dissipates to the environment in the cooling tower.

Post-harvest storage of most fruits and vegetables requires an atmosphere of high relative humidity to prevent the physiological loss in weight and shrinkage of the crops. For maintaining higher humidity inside the cooling chamber, a Rivulis Irrigation Fogger of the pressure range of 50–70 psi was installed inside the chamber and operated by a diaphragm pump (12 V DC) powered by an electric battery.

The developed refrigeration system can attain cooling temperature in the range of 15–17 °C and relative humidity of 80–90% when loaded with 12 kg of fruits and vegetables and at an ambient temperature in the range of 35 to 40 °C. The detailed specifications of the developed refrigeration system have been presented in Table 1.

### 2.2. Vapour Compression Refrigeration System

To compare the developed thermoelectric refrigeration system with commercially available refrigeration technology, a vapour compression refrigerator (Godrej and Boyce Mfg Co Ltd., Mumbai, India) of 150-L capacity, operated using isobutene refrigerant and powered by 230 V power, was used in the present study.

### 2.3. Storage Stability and Quality of Fruits and Vegetables

The performance of the developed mobile thermoelectric refrigeration system was evaluated based on the storage stability of fruits and vegetables. The freshly harvested summer fruits and vegetables (mango, papaya, bitter gourd, okra) were procured from the Punjab Agricultural University farm. A 3-kg sample of each crop was stored at thermoelectric refrigeration (temp 15–18 °C and RH 80–90%), vapour compression refrigeration (temp 6–8 °C and RH 80–90%), and ambient atmospheric (temp 35–40 °C and RH 40–50%) storage conditions, respectively. Every day, five crops from each storage condition were selected randomly for quality analysis. The storage stability of the commodities stored in the developed refrigeration system was compared with the vapour compression refrigeration system and under ambient environmental conditions. The system was evaluated based on physiological loss in weight, texture, colour, and physical appearance of the commodity stored in the system. The texture and colour of each crop were measured at three different locations. The changes in the quality parameters of stored fruits and vegetables were evaluated daily. The procedure followed for the evaluation of quality parameters is explained next.

#### 2.3.1. Physiological Loss in Weight (PLW)

Physiological loss in weight is the reduction in weight of a commodity due to physicochemical reactions taking place in food over a period of time. The physiological loss in weight was determined as per the method described by [26].

$$\mathrm{Physiological}\;\mathrm{loss}\;\mathrm{in}\;\mathrm{weight}\;\mathrm{(PLW)}\;=\;\frac{W_{1}-W_{2}}{W_{1}}\;\times 100$$

where

W_1_—Weight of sample on 0th day;

W_2_—Weight of sample on nth day.

#### 2.3.2. Determination of Firmness

Firmness is one of the parameters of texture analysis that measures the force required to penetrate a stainless-steel probe inside the food crop and is measured in Newton force (N). The firmness of okra and bitter gourd was measured with the help of a texture analyzer machine (Make: Instron, TA-HDi). The compression test was performed using a 2-mm stainless-steel probe [27]. The machine operating parameters selected for this test were:
Contact force—100 gPre-test speed—1.0 mm/sTest speed—2.0 mm/sCompression distance—10.0 mmPost-test speed—10.0 mm/sReturn speed—5 mm/sReturn distance—20 mm


#### 2.3.3. Measurement of Colour Attributes

The colour attributes of the crop i.e., ‘*L*’, ‘*a*’ and ‘*b*’, were recorded using a colour reader instrument, CR-10 (Konick Minolta Sensing Inc. Japan), on the surface of the crop at three different locations. The total change in colour (∆E) attributes over the period of storage time was calculated by the following equation [26]:

$$\Delta E=\sqrt{{\left(L_{0}-L_{t}\right)}^{2}+{\left(a_{0}-a_{t}\right)}^{2}+{\left(b_{0}-b_{t}\right)}^{2}}$$

where *L*_0_, *a*_0_, *b*_0_ are the initial colour measurements of the 0th day, and *L_t_*, *a_t_*, *b_t_* are the colour measurements at a pre-specified time.

#### 2.3.4. Overall Acceptability of the Crop

The sensory evaluation scale for rating the overall acceptability of the stored fruits and vegetables was developed based on three main parameters, i.e., appearance, colour, and texture. The sensory assessment for the samples was carried out during natural day-light by a five-member (3 male and 2 female) trained panel in the age group of 35 to 50 years, and these quality parameters of the samples were examined by using the rating scales proposed respectively by [28]. All the samples were arranged for visual quality evaluation and samples were ranked using a nine-point heddonic scale: 1—dislike extremely, 2—dislike very much, 3—dislike moderately, 4—dislike slightly, 5—neither dislike nor like, 6—like slightly, 7—like moderately, 8—like very much, and 9—like extremely. At the end of the session, panellists were asked to report their purchase intention on the following scales: 9—definitely would buy, 7—probably would buy, 5—maybe/maybe not, 3—probably would not buy, and 1—definitely would not buy [29].

### 2.4. Statistical Analysis

All the experimentation was carried out in triplicate. The data obtained during the quality evaluation of fruits and vegetables were subjected to a two-way analysis of variance (ANOVA) using SAS statistical software (SAS 9.4 SAS Institute for data management, sourced from PAU, Ludhiana, India). Significance differences between average rates of change of quality parameters of crops stored in thermoelectric refrigeration, vapour compression refrigeration and ambient storage conditions were resolved by Tukey’s test for comparison. A *p* < 0.05 value indicated the significance of the difference in the effect of storage condition and crop species on quality parameters during storage.

## 3. Results and Discussion

### 3.1. Effect of Storage System on Physiological Loss in Weight (PLW)

The PLW is the key element for shelf life and post-harvest quality evaluation of fruits and vegetables. It is the measure of loss in the weight of a crop over a period of time due to physicochemical changes in the crop. The loss in weight of fruits and vegetables stored in LCTR, VCR, and at ambient conditions was observed. It was observed that weight loss of all four crop species, i.e., bitter gourd, okra, mango, and papaya, increased with an increase in storage period irrespective of storage condition and crop species. Among the vegetables, crops stored in the VCR witnessed the least change in PLW of 13.58% and 12.90% for bitter gourd and okra at the end of 9 and 10 days of storage, respectively. Conversely, the highest PLW of 16.83% and 17.71% was recorded for crops stored in ambient storage conditions at the end of 4 and 5 days of the storage period. The PLW of crops stored in LCTR lied at intermediate as 11.51% and 10.99% for bitter gourd and okra after 7 and 8 days of storage, respectively. The ambient storage condition reported the highest average rate of change of PLW, followed by LCTR and VCR storage for bitter gourd as well as okra. Among the fruits, the least PLW was recorded at ambient storage as 10.33% and 8.15% for mango and papaya at the end of 6 and 4 days, respectively. The highest PLW was reported in the VCR system for mango (21.22%) and papaya (16.52%), respectively, at the end of the storage period. The LCTR system reported intermediate PLW of 12.29% and 19.17% for mango and papaya, respectively. Though the VCR system registered the highest increase in PLW, it reported the least average rate of change in PLW, followed by LCTR and ambient storage for mango as well as papaya, as shown in Table 2.

**Figure 4:**
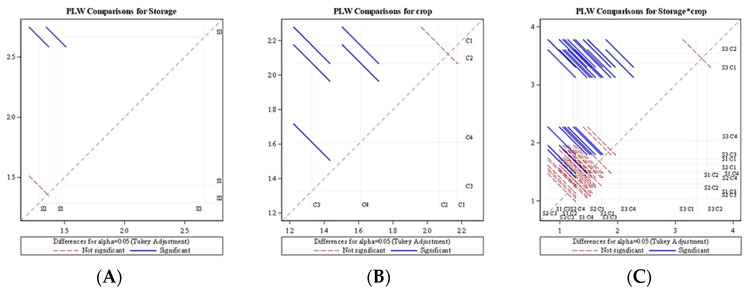
Diffogram showing the effect of storage condition and crop on the average daily change of PLW. (**A**) PLW comparison for storage (**B**) PLW comparison for crop (**C**) PLW comparison for storage*crop (S1—LCTR; S2—VCR; S3—ambient storage; C1—bitter gourd; C2—okra; C3—mango; C4—papaya).

An analysis of variance (ANOVA) for the average change of PLW indicated a significant effect of the different storage systems (*p* < 0.0001) as well as crop species (*p* < 0.0001). The diffogram generated from the ANOVA indicated that no significant difference in the PLW value existed between the LCTR and VCR system, as the red line of the LCTR and VCR passed through the confidence interval, whereas the ambient storage condition was significantly different from the LCTR and VCR systems (Figure 4). Similarly, the red line of bitter gourd and okra passing through the confidence interval indicated that no significant difference existed among the vegetables. However, significant differences existed among fruits, i.e., mango and papaya, as well as between fruits and vegetables. It was also observed that the interaction of the storage system and crop species had a significant effect (*p* < 0.0001) on physiological loss in the weight of the crop (Table 2).

The overall increase in the PLW with the increase of storage duration was attributed to respiration by the crop species, transpiration of water through the tissues, and other physicochemical changes taking place in the crop [30,31]. As the fresh produce continues to respire during storage, carbon loss through the gas exchange during the respiration process causes a reduction in the weight of the crop, while transpiration of water vapour from the surface of the crop was another contributor to PLW from the crop [32]. The metabolic activities of the fruits and vegetables continue after the harvest of the crop. The difference in water vapour pressure at the surface of the product and the environment causes moisture loss from the product, which leads to a reduction in the weight of the product [33]. Hence, the physiological loss in the weight of the crop is equivalent to the loss of water vapour from the surface of the crop and loss of carbon during respiration of the crop [32]. Similar results were reported by Mohammed and Wickham [34] for bitter gourd wrapped with LDPE packaging and stored at different temperatures.

### 3.2. Effect of Storage Condition on Firmness

The texture of vegetables has been considered as an index for consumer acceptability of commodity, as it determines the quality and freshness of the crop. The firmness of the fruits and vegetables depends upon the physical anatomy of the tissue, i.e., cell size and shape, strength, the way in which cells bind together to form a tissue, and wall thickness [35,36].

It was observed that the firmness of bitter gourd and okra decreased with an increase in storage duration, irrespective of the storage system. The highest loss in firmness of 41.2% and 47.8% were registered under the ambient storage system at the end of 4 and 5 days of storage for bitter gourd and okra, respectively. On the contrary, the highest retention of firmness was recorded for crops stored in the VCR system, which reduced by 52.7% and 46.3% for bitter gourd and okra at the end of 9 and 10 days of storage life, respectively. The firmness retention capacity of the LCTR system was comparable with the VCR system at 58.7% and 41.9% at the end of 7 and 8 days of storage for bitter gourd and okra, respectively.

**Figure 5:**
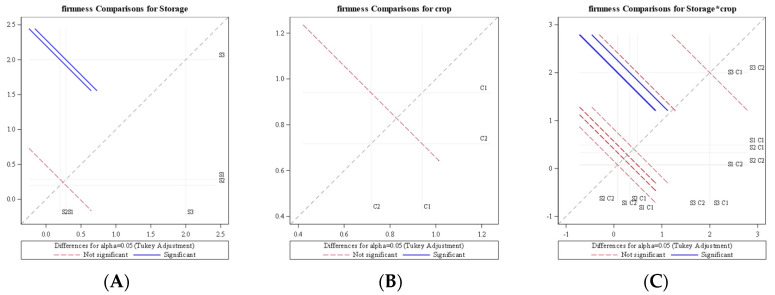
Diffogram showing the effect of storage condition and crop on average daily firmness reduction. (**A**) Firmness comparison for storage (**B**) Firmness comparison for crop (**C**) Firmness comparison for storage*crop S1—LCTR; S2—VCR; S3—ambient storage; C1—bitter gourd; C2—okra.

An analysis of variance (ANOVA) for the average rate of firmness reduction indicated the significant effect of the different storage systems (*p* = 0.0002), whereas a nonsignificant effect existed between bitter gourd and okra (*p* = 0.430) at a 5% level of significance. The diffogram generated from ANOVA (Figure 5) indicated that no significant difference existed between LCTR and VCR; however, the ambient storage system was significantly different from LCTR and VCR. It was also revealed from the diffogram that average daily firmness reduction was the same for bitter gourd and okra irrespective of the storage system, as seen by the interaction line passing through the region of the confidence interval. It was also observed that the interaction of the storage system and crop had no significant effect (*p* = 0.828) on the firmness of the crop, as presented in Table 2.

The decreasing trend of firmness with the increasing storage period of the crop was attributed to the mechanism of cell wall digestion by pectinesterase, polygalacturonase, and other enzymes. The process of the disruption of the tissue and cell wall accelerates in crops stored at higher temperatures [37]. The study conducted by Manjunatha and Anurag [11] on the storage of cucumbers at different temperatures and relative humidity also showed the negative effect of storage duration on the firmness of cucumbers. The maximum retention of firmness of the crop stored at lower temperature was attributed to the presence of higher relative humidity, which prevents the transmission of moisture and ultimately shrinkage of the crop. Jin et al. [38] explained the mechanism of firmness retention at a lower temperature as a halt of enzyme activities, delayed oxidative decomposition, and inhibition of expression of many genes during cold storage. The reduction in firmness at higher storage temperatures was due to the breakdown of insoluble protopectin into soluble pectin and cellular disintegration [39]. Mohammed and Wickham [34] also reported a loss in firmness of bitter gourd during storage due to shrinkage of the product caused by water loss.

### 3.3. Effect of Storage Condition on Colour

Colour is one of the major visual attributes of fruits and vegetables. It is the colour of the commodity that comes into the first appearance and influences consumers’ attraction toward crops. The surface colours of okra, bitter gourd, mango, and papaya were measured to the determination of changes in colour due to physiochemical reactions and ripening of fruits and vegetables during storage. For the determination of the effect of storage conditions and storage period on the colour of the crop, colour attributes i.e., ‘a’, and colour change ‘∆E’ were determined and explained under the following sub-headings.

#### 3.3.1. Effect of Storage Condition on Colour Value ‘a’

The colour value ‘a’ varies from negative to positive representing the greenness to redness of the crop. The greenish appearance of fruits and vegetables is the indicator of chlorophyll content in crops [35].

**Figure 6:**
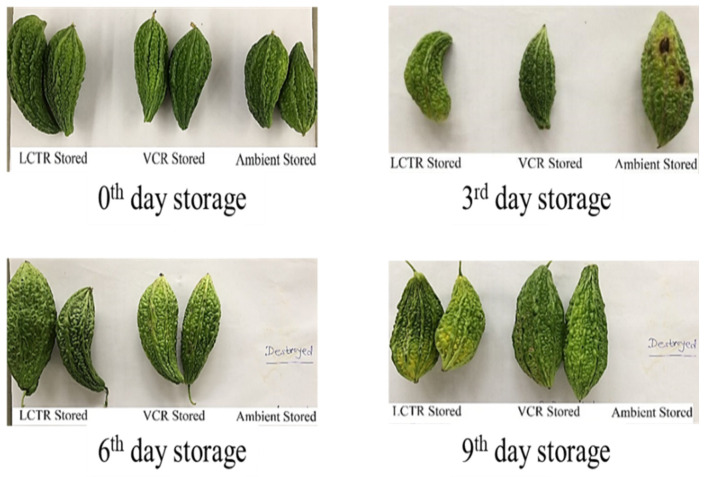
Effect of storage system on the visual appearance of bitter gourd.

**Figure 7:**
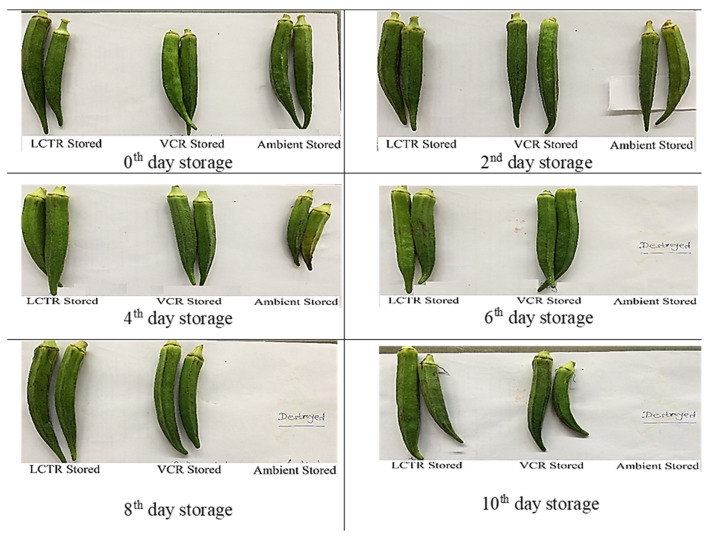
Effect of storage system on the visual appearance of okra.

**Figure 8:**
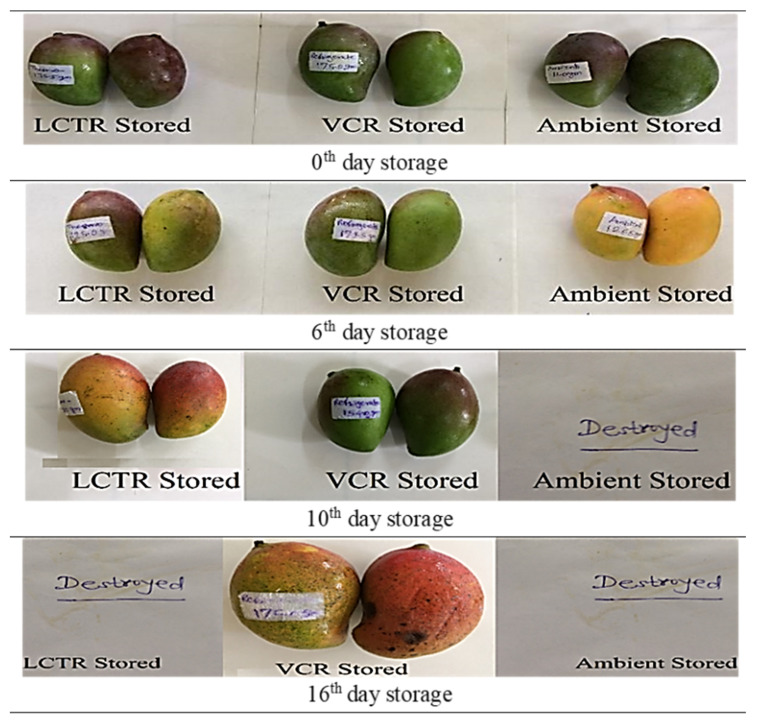
The visual appearance of mango during storage.

**Figure 9:**
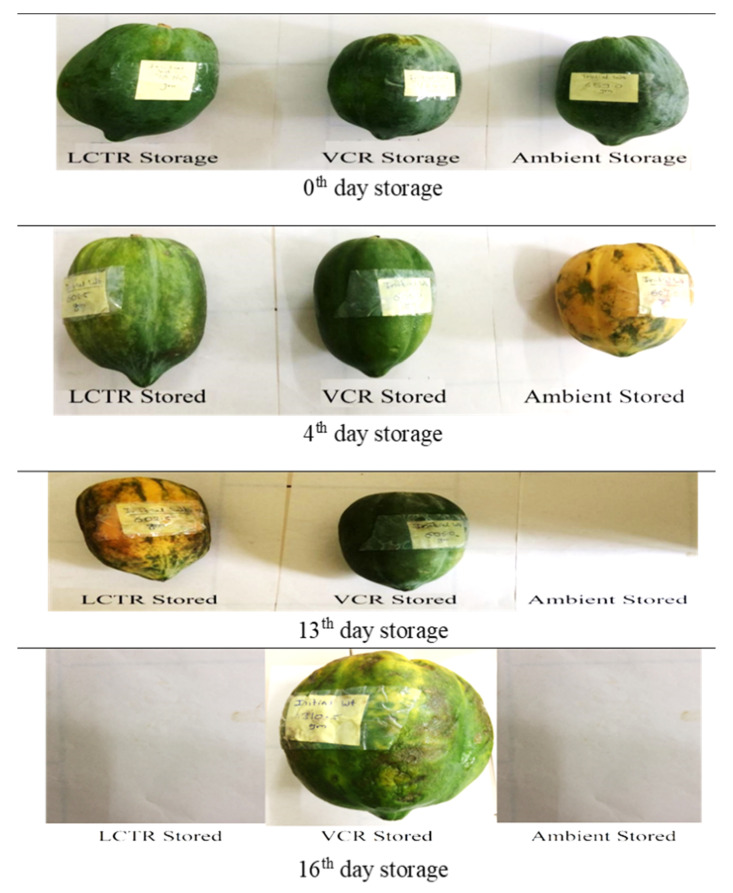
The visual appearance of papaya during storage.

In the present study, ‘-a’ represented the green value of all the crops deteriorated during the storage period irrespective of storage conditions. A decreasing trend of colour value ‘-a’ for all the varieties was revealed from the graphical presentation for bitter gourd, okra, mango, and papaya in Figure 6, Figure 7, Figure 8 and Figure 9, respectively. Among the vegetables, a decrease of the ‘a’ value was more pronounced in ambient conditions for bitter gourd (43.33%) and okra (48.53%) and least in VCR for bitter gourd (24.17%) and okra (28.67%). The greenness retention capacity of LCTR lay intermediate to that of ambient and VCR storage. The (-a) value of crops stored in the LCTR declined by 38.33% and 31.62% for bitter gourd and okra during the storage period, respectively. Similarly, among the fruits, the decrease in lightness was more pronounced for fruits stored in ambient storage for mango (50.00%) and papaya (65.77%) at the end of four and six days of storage, respectively, whereas the least change was recorded in VCR storage of mango (47.52%) and papaya (64.43%) at the end of 16 days of storage. The LCTR storage system reported an intermediate effect of greenness reduction, recording 47.30% and 64.47% reductions for mango and papaya at the end of 10 and 13 days of storage, respectively.

**Figure 10:**
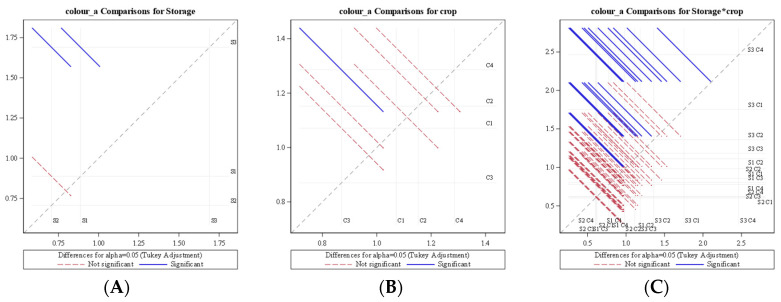
Diffogram showing the effect of storage condition and crop on average daily change of colour value a. (**A**) Colour ‘a’ comparison for storage (**B**) Colour ‘a’ comparison for crop (**C**) Colour ‘a’ comparison for storage*crop S1—LCTR; S2—VCR; S3—ambient storage; C1—bitter gourd; C2—okra; C3—mango; C4—papaya.

An analysis of variance (ANOVA) for average daily change of colour value ‘a’ revealed that colour value ‘a’ was significantly influenced by storage condition (*p* < 0.0001) as well as crop species (*p* = 0.0091) at a 5% level of significance. The diffogram (Figure 10) generated from ANOVA also indicated that no significant difference of average daily change of colour value ‘a’ existed among LCTR and VCR storage conditions, whereas the ambient storage condition was significantly different from LCTR and VCR storage. The diffogram also revealed that the average rate of reduction in greenness for mango was significantly different from that of papaya, whereas no significant difference existed between mango, bitter gourd, and okra. It was also observed from Table 2 that the interaction of storage condition and crop had a significant effect (*p* < 0.0001) on the rate of decrease in lightness value.

The reduction of the ‘a’ value was in accordance with Devi et al. [40]. It was also reported that storage of green crops at higher temperatures resulted in the rapid transition of crop colour from green to reddish. The change in colour of the crop was due to the ripening of the crop. It was also reported that storage of the green crop at a higher temperature resulted in a rapid transition of crop colour from green to yellowish. The increase in yellowness during the storage period might be due to the breakdown of chlorophyll, as well as the increase in carotenoid inside the pulp during physico-chemical changes taking place during ripening, which leads to the turning of the green colour of the crop from light green or dark green to yellowish or yellowish-red [41].

#### 3.3.2. Effect of Storage Condition on Overall Colour Change

The colour change value (∆E) represents an overall change in the colour of the crop during the storage period compared to the initial colour of that crop. In the present study, the increasing trend of the colour change value was observed during the storage period, irrespective of storage conditions and crop species.

Among the vegetables, ambient storage conditions had a remarkable effect on overall colour change (∆E) for bitter gourd (21.97) and okra (18.48), whereas the least colour change was recorded in VCR storage for bitter gourd (15.02) and okra (13.81) at the end of the respective storage duration. The colour change for crops stored in the LCTR system was intermediate between ambient and VCR storage. The colour change for LCTR storage was 14.04 and 11.46 for bitter gourd and okra at the end of the storage period. Similarly, among the fruits, ambient storage conditions reported the highest increase in colour value for both fruits, which was reported to be 21.89 and 20.60 for mango and papaya, respectively, at the end of their respective storage period. The least colour change was recorded for fruits stored in VCR storage conditions, registering 18.42 and 21.77 for mango and papaya, respectively. The LCTR storage system reported an intermediate effect on colour change, recording 16.41 and 23.68 for mango and papaya, respectively, at the end of the storage duration.

**Figure 11:**
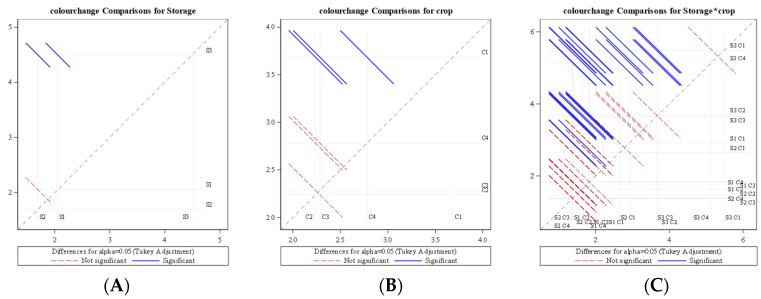
Diffogram showing the effect of storage condition and crop on average daily colour change. (**A**) Colour change comparison for storage (**B**) Colour change comparison for crop (**C**) Colour change comparison for storage*crop S1—LCTR; S2—VCR; S3—ambient storage; C1—bitter gourd; C2—okra; C3—mango; C4—papaya.

It was also revealed from the analysis of variance (ANOVA) for the average rate of change of colour (∆E) that storage condition, as well as crop species, had a significant effect (*p* < 0.0001) on colour change at a 1% level of significance. The diffogram (Figure 11) generated from ANOVA indicated that no significant difference in average daily change of colour existed among LCTR and VCR storage conditions, whereas the ambient storage condition was significantly different from LCTR and VCR storage. The diffogram also revealed that the average daily change of colour change for bitter gourd was significantly different from all the other crops, while no significant difference existed among okra, mango, and papaya. It was also observed from Table 2 that the interaction of storage condition and the crop had a significant effect (*p* < 0.0489) on the rate of increase in colour change value.

The change in colour during the storage of fruit and vegetables was also reported by [31,42,43]. It was also reported that the storage of green crops at higher temperatures resulted in the rapid transition of crop colour from green to yellowish. The increase in yellowness during the storage period might be due to the breakdown of chlorophyll, as well as an increase in carotenoid inside the pulp during physicochemical changes taking place during ripening, which leads to the turning of the green colour of the crop from light green or dark green to yellowish or yellowish-red [38]. The change in colour from green to yellow of mango and papaya was due to chlorophyll degradation and carotenoid synthesis during the maturity of fruits.

### 3.4. Effect of Storage Condition on Overall Acceptability

The overall acceptability of fruits and vegetables is a vital parameter, as it directly determines the consumer’s perception of the quality and willingness to purchase the crop. The acceptability of the crop was evaluated in terms of colour changes, visual spoilage, softness, and ripeness.

It was observed that the overall acceptability showed a decreasing trend during storage, irrespective of storage conditions and crop species. The changes in visual appearance of bitter gourd, okra, mango, and papaya during the storage period are presented in Figure 6, Figure 7, Figure 8 and Figure 9 respectively. Among the vegetables, the highest reduction in overall acceptability was witnessed for crops stored at ambient storage conditions, which recorded decreases in acceptability from 9.0 to 3.3 and from 9.0 to 4.0 at the end of 4 and 5 days of storage for bitter gourd and okra. Conversely, crop stored in VCR was acceptable for a longer duration, which decreased from 9.0 to 5.3 and from 9.0 to 5.3 for bitter gourd and okra for the respective storage duration. The acceptability of crops stored in LCTR was intermediate, which decreased from 9.0 to 5.0 and 9.0 to 5.3 for bitter gourd and okra, respectively. Similarly, among the fruits, the ambient storage condition reported the highest decrease in the average acceptability for both fruits, which decreased from 9.0 to 5.0 and from 9.0 to 5.0 for mango and papaya, respectively, during the storage period. The least decrease in acceptability was recorded for fruits stored in VCR storage conditions, registering an increase from 9.0 to 5.0 and from 9.0 to 5.0 for mango and papaya, respectively. The LCTR storage system reported an intermediate effect on acceptability, recording a decrease from 9.0 to 5.0 and from 9.0 to 4.7 for mango and papaya, respectively, during the storage duration.

**Figure 12:**
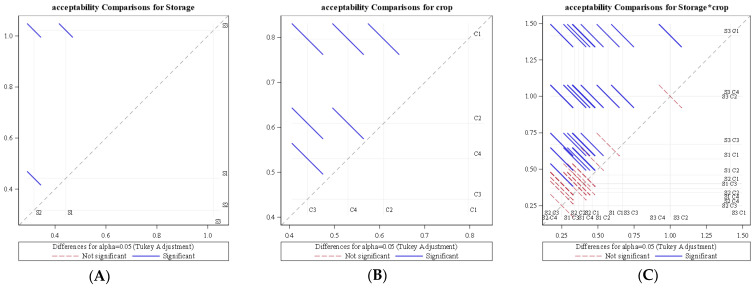
Diffogram showing the effect of storage condition and (**B**) crop on average daily change of overall acceptability. (**A**) Acceptability comparison for storage (**B**) Acceptability comparison for crop (**C**) Acceptability comparison for storage*crop S1—LCTR; S2—VCR; S3—ambient storage; C1—bitter gourd; C2—okra; C3—mango; C4—papaya.

An analysis of variance (ANOVA) for the average rate of change of overall acceptability revealed that acceptability was significantly influenced by storage condition (*p* < 0.0001) as well as crop species (*p* < 0.0001) at a 1% level of significance. The diffogram (Figure 12) generated from the ANOVA also indicated that there was a significant difference among LCTR, VCR, and ambient storage conditions for the rate of change of the overall acceptability of the crop. The diffogram also revealed that significant differences existed among bitter gourd, okra, mango, and papaya for the average rate of change of acceptability. It was also observed that the interaction of storage condition and crop species had a significant effect (*p* < 0.0001) on the rate of decrease of acceptability. Table 2 indicates the variation in the average rate of decrease in acceptability for the interaction of storage conditions and crop species.

Based on the score assigned to crops on a daily basis and visual appearance, it was concluded that bitter gourd stored in LCTR, VCR, and ambient conditions had an acceptability of 7, 9, and 3 days, respectively. Similarly, the acceptability of okra, mango, and papaya were as follows; okra (LCTR 8; VCR 10; ambient store 4), mango (LCTR 10; VCR 16; ambient store 6), and papaya (LCTR 12; VCR 16; ambient store 4). The decrease in acceptability was due to the growth of fungus and blackish colour in bitter gourd and okra, and the ripening of mango and papaya, whereas crops stored in LCTR and VCR were in an enclosed chamber and under hygienic conditions, which prevented the external attack from microorganisms.

## 4. Conclusions

An eco-friendly thermoelectric refrigerator of 100-litre capacity based on the Peltier effect was developed, and the performance was evaluated for keeping the quality of fruits and vegetables as compared to the same stored at ambient temperature. The thermoelectric refrigerator can be operated at the temperature range of 15–17 °C and relative humidity of 80–90%, which was approaching the storage conditions required for most summer fruits and vegetables. The thermoelectric refrigeration facilitated impressive results for the quality retention of fruits and vegetables during storage. A remarkable improvement in the shelf life of all the fruits and vegetables was reported by using thermoelectric refrigeration in comparison to the ambient storage system. The shelf life of bitter gourd, okra, mango, and papaya stored in a thermoelectric refrigeration system was increased up to 7, 8, 10, and 12 days, respectively. As the statistical analysis indicated no significant difference in the quality attributes of a commodity stored in the vapour compression refrigeration and thermoelectric refrigeration systems, it was concluded that adequate scope exists for the utilization of the developed thermoelectric refrigeration system for the cold storage of fruits and vegetables. As there is a lack of scientific cold storage infrastructure for maintaining the quality of fruits and vegetables during transportation in rural areas and being an eco-friendly and sustainable refrigeration technology, the thermoelectric refrigerator is not only helpful for generating carbon credits but also facilitates cold chain management of perishable crops. The developed LCTR system is useful for the on-field pre-cooling of freshly harvested fruits and vegetables and cold chain management during the transportation of perishable crops from field to storage house. The system is also helpful to vegetable vendors for the cold and hygienic storage of fruits and vegetables during marketing. Effective cold chain techniques at the community level act as a link between the agriculture field and end consumer by stretching the marketable time of the perishable products. The producers can also meet the demands and have a higher share of profit with reduced loss. Farmers and owners of the produce can reach newer markets to realize greater circular economic value.

## Figures and Tables

**Table 1:** Specifications of the liquid-cooled thermoelectric refrigeration system.

Sr. No.	Particular	Specification
1	Overall dimension of LCTR	145 × 650 × 120 cm
2	Material of cooling chamber	Polyurethane
3	Cooling capacity of LCTR	0.31 KW
4	Dimension of cooling chamber	45 × 50 × 50 cm
5	Cold chamber storage capacity	100 L
6	Peltier module	TEC 12706 A; (12 V DC, 6 A)
7	Number of Peltier modules	4
8	Number of water-cooled heat sink	4
9	Number of air-cooled heat sink	3
10	Number of water pump	2
11	Number of fogger	1
12	Electric battery	1 (160 Ah)
13	Water tank capacity (l)	15
14	Cooling capacity	Temp 15–17 °C/RH 80–90%
15	Coefficient of performance (COP)	0.85

**Table 2:** Statistical significance for the interaction of storage method and crop for average daily changes in quality parameters of the crop.

Crop	Storage Method	Quality Parameters				
		PLW	Firmness	Colour Value (a)	Colour Change (ΔE)	Overall Acceptability
Bitter Gourd	LCTR	1.643 ^cbd^	0.489 ^ab^	0.857 ^cd^	2.917 ^bc^	0.57 ^cd^
	VCR	1.507 ^cde^	0.330 ^b^	0.603 ^d^	2.647 ^bcd^	0.40 ^ef^
	Ambient	3.367 ^a^	2.000 ^a^	1.753 ^b^	5.490 ^a^	1.42 ^a^
Okra	LCTR	1.377 ^cde^	0.080 ^b^	1.113 ^bcd^	1.637 ^d^	0.46 ^de^
	VCR	1.290 ^cde^	0.071 ^b^	0.983 ^cd^	1.383 ^d^	0.37 ^ef^
	Ambient	3.543 ^a^	2.000 ^a^	1.360 ^bc^	3.697 ^b^	1.00 ^b^
Mango	LCTR	1.230 ^de^	NA	0.803 ^cd^	1.837 ^cd^	0.40 ^ef^
	VCR	1.033 ^e^	NA	0.623 ^d^	1.373 ^d^	0.25 ^f^
	Ambient	1.720 ^bc^	NA	1.183 ^bcd^	3.647 ^b^	0.67 ^c^
Papaya	LCTR	1.473 ^cde^	NA	0.777 ^cd^	1.823 ^cd^	0.34 ^ef^
	VCR	1.323 ^cde^	NA	0.617 ^d^	1.370 ^d^	0.25 ^f^
	Ambient	2.037 ^b^	NA	2.463 ^a^	5.150 ^a^	1.00 ^b^

Same lowercase superscript letters (a, b, c, …) in column indicate no statistically significant difference at 95% of the confidence interval. PLW: Physiological Loss in Weight.

## Data Availability

Data is contained within the article.
